# Transcriptomic analyses of *Vibrio parahaemolyticus* under the phenyllactic acid stress

**DOI:** 10.1007/s00253-024-13024-6

**Published:** 2024-01-29

**Authors:** Yilin Lin, Meimei Fang, Jun Liu, Yehui Zhang, Yigang Yu

**Affiliations:** 1 South China University of Technology, School of Food Sciences and Engineering, Guangzhou, 510640 China; 2 Sericulture & Agri-Food Research Institute Guangdong Academy of Agricultural Sciences, Key Laboratory of Functional Foods, Ministry of Agriculture and Rural Affairs, Guangdong Key Laboratory of Agricultural Products Processing, Guangzhou, 510610 China; 3 South China University of Technology, Research Center of Food Safety and Detection, Guangzhou, 510640 China

**Keywords:** *Vibrio parahaemolyticus*, Phenyllactic acid, Antibacterial, Transcriptomics

## Abstract

**Abstract:**

Phenyllactic acid (PLA) generally recognized as a natural organic acid shows against *Vibrio parahaemolyticus* activity. In this study, *V. parahaemolyticus* ATCC17802 (*Vp*17802) was cultured under the stress of 1/2MIC PLA, and then the antibacterial mechanisms were explored via transcriptomics. The minimum inhibitory concentration (MIC) of PLA against *Vp*17802 was 3.2 mg/mL, and the time-kill analysis resulted that *Vp*17802 was inhibited. PLA was able to destroy the bacterial membrane, leading to the leakage of intracellular substances and decline of ATP levels. The RNA-sequencing analysis results indicated that 1616 significantly differentially expressed genes were identified, among which 190 were up-regulated and 1426 were down-regulated. Down-regulation of the *icd2* gene in the TCA cycle mediates blockage of tyrosine metabolic, arginine biosynthesis, and oxidative phosphorylation, causing insufficient energy supply of *Vp*17802. Moreover, PLA could cause amino acids, metal ions, and phosphate transporters to be blocked, affecting the acquisition of nutrients. The treatment by PLA altered the expression of genes encoding functions involved in quorum sensing, flagellar assembly, and cell chemotaxis pathway, which may be interfering with the biofilm formation in *Vp*17802, reducing cell motility. Overall, 1.6 mg/mL PLA inhibited the growth of *Vp*17802 by disrupting to uptake of nutrients, cell metabolism, and the formation of biofilms. The results suggested a new direction for exploring the activity of PLA against *Vp*17802 and provided a theoretical basis for bacterial pathogen control in the food industry.

**Key points:**

*•RNA sequencing was carried out to indicate the antibacterial mechanism of Vp17802.*

*•The icd2 gene in the TCA cycle mediates blockage of metabolic of Vp17802.*

*•The biofilm formation has interfered with 1.6 mg/mL PLA, which could reduce cell motility and virulence.*

**Graphical Abstract:**

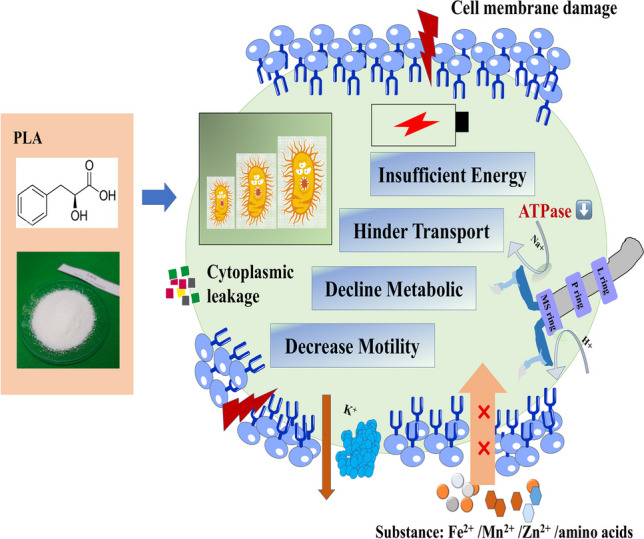

**Supplementary Information:**

The online version contains supplementary material available at 10.1007/s00253-024-13024-6.

## Introduction

Food safety problems are becoming increasingly prominent due to foodborne pathogenic contamination. *Vibrio parahaemolyticus* is a gram-negative halophilic bacterium, which is mainly distributed in the marine environment, and widely exists in fresh aquatic products such as *Penaeus vannamei* (Feng et al. 2021), *oyster* (Mohamad & Li 2022) and raw *salmon* fillets (Fang et al. 2021). *V. parahaemolyticus* has a strong pathogenic ability that can cause watery diarrhea, abdominal convulsions, nausea, vomiting, and headaches in humans, leading to sepsis and even death in severe cases (Li et al. 2019). According to the Disease Control and Prevention Center, this bacterium causes 215 foodborne disease cases per year, causing food poisoning or life threats (Elmahdi et al. 2016). Wu et al. (2014) have reposed that *V. parahaemolyticus* caused 322 foodborne outbreaks from 2003 to 2008 in China, and the number of patients reached 9041. In Japan, *V. parahaemolyticus* was also associated with 20–30% of human food poisoning (Jahangir et al. 2002).

The pathogenesis mechanism of *V. parahaemolyticus* is mainly related to its virulence factor, including two types III secretion systems (T3SS1 and T3SS2), adhesion factor, flagellar system, biofilm, and heat-resistant direct hemolysin (TDH). T3SS1 releases essential nutrients to ensure the survival of *V. parahaemolyticus*, and T3SS2 or TDH can release different virulence factors leading to different pathogenicity strains (Makino et al. 2003). There is a complex regulatory network in virulence factors. Adhesion factors, flagellar system, and biofilm mediate the contact between bacteria and host, and the secretion system mediates the secretion of effector proteins to induce invasion of host cells.

Recently, many natural antimicrobial agents from plant, animal, or microbial fermentation have been developed due to the insecurity of synthetic antibacterial substances and the increasing demand of consumers (Juneja et al. 2012). Phenyllactate acid (PLA) was first discovered from *Geotrichum candidum* by Dieuleveux in 1998 isolated from a metabolite (Dieuleveux & Guéguen 1998). PLA is a natural organic acid with broad antibacterial activity widely found in fermented goods, metabolites of lactic acid bacteria, and natural plants (Nhung et al. 2017). It exhibits inhibitory effects on a wide range of bacteria, including *Listeria monocytogenes* (Dieuleveux & Guéguen 1998), *Salmonella* (Rodríguez et al. 2012), and *Escherichia coli* O157:H7 (Zheng et al. 2019). The hydrophobic benzene ring and hydrophilic carboxyl structure confer amphiphilicity on PLA, which may facilitate cross-linking with bacterial membrane lipids and membrane proteins. Furthermore, PLA has water solubility and non-toxicity, which can stimulate active immune cells to show anti-inflammatory effects in the human body (Peters et al. 2019). It has been reported that PLA has heat stability whose antimicrobial activity is unaffected even at 121 °C/20 min (Cortes et al. 2014), which can enhance the shelf-life of food. However, the activity of PLA is greatly affected by acidity, and lower pH conditions can enhance the antimicrobial activity of PLA. We previously found that 1/2MIC PLA significantly reduced the CFU of *Vp*17802 on the salmon during the 8-day storage, and the antibacterial activity of PLA was found to be in a dose-dependent manner (Feng et al. 2021).

The antibacterial mechanism of PLA mainly focuses on the cellular level, including cell membrane integrity destruction and genomic DNA degradation (Fang et al. 2017). Dieuleveux and Guéguen (1998) have revealed that the antibacterial mechanism of PLA is likely associated with destroying the cell wall, and Ning et al. (2020) have reported that PLA can inactivate the cell membrane integrity of *Listeria monocytogenes*, while for *Escherichia coli*, it only destroys the outer membrane. The active sites of PLA on gram-positive bacteria and gram-negative bacteria are different. Recent evidence suggests that energy metabolism disorder is a new target of PLA inhibition of *Rhizopus oryzae *(Fan et al. 2022). PLA has been recognized as a natural, safe, and green antibacterial substance in the food industry, a deeper understanding of the subcellular targets and antibacterial mechanisms of PLA were required for their practical applications.

Therefore, transcriptome sequencing (RNA-seq) further clarifies the antibacterial mechanism of PLA in *V. parahaemolyticus*, which plays a vital role in cell growth and proliferation, promoting their application in the food industry.

## Materials and methods

### Bacterial strain and chemical

*V. parahaemolyticus* ATCC 17802 (*Vp*17802) was obtained from the Gongbei Customs Technical Center (Zhuhai, Guangzhou), and it was stored in tryptic soy broth (TSB) supplied with 3% NaCl (TSB-s) and 25% glycerol at − 80 °C before use. The bacterium was activated by incubation at 37 °C for 24 h after streaking the stock culture onto tryptone soy agar (TSA) supplied with 3% NaCl (TSA-s, pH 7.3). Then, a single colony from TSA-s was inoculated into TSB-s to reach the stationary phase after incubation at 37 °C for 18 h. Stock solutions of phenyllactate acid (PLA, 98%; Aladdin Reagent Co., Ltd., Shanghai, China) was prepared just before use and briefly stored at 4 °C.

### Determination of minimum inhibitory concentration and time-kill analysis

The minimum inhibitory concentration (MIC) of PLA against *Vp*17802 was determined using broth microdilution method described previously (Fang et al. 2021). The time-kill assay was carried out to measure the bactericidal effect of PLA against *Vp*17802. The *Vp*17802 was diluted to about 10^6^ CFU/mL in the TSB-S containing PLA with the final concentrations of 0.8 mg/mL, 1.6 mg/mL, 3.2 mg/mL, and 6.4 mg/mL respectively. The sterile TSB-s without PLA was used as the control group, and all samples were incubated under microaerophilic conditions with treatment for 0 h, 4 h, 8 h, 12 h, 16 h, 20 h, and 24 h. The viable bacteria were calculated by plate counting.

### Determination of cytoplasmic leakages

The bacterial suspension was filtered through a membrane of 0.22 μm pore size. The filtrate was collected, and its absorbance at 260 nm and 280 nm was determined using an ND2000C ultra-micro ultraviolet–visible spectrophotometer (Thermo Fisher Scientific (China) Co., Ltd.).

### Determination of ATPase activity and ATP level

The ATPase activity was determined using an ultra-micro total ATPase test kit (Nanjing Jiancheng Bioengineering Institute, A070-1), and the ATP concentration was determined using an ATP test kit (Biyuntian Biotechnology, S0026).

### Swimming test

The final concentration of 1/4 MIC and 1/2 MIC PLA were added to the TSB-S which contains 0.2% agar as the treated group, and an equal volume of sterile water as the control group. All plates were incubated at 37 ℃ for 12 h to use for swimming tests.

### RNA isolation and processing

Two sets of samples were selected for transcriptomic analyses to explore the antibacterial mechanisms of PLA. The bacterial suspensions were treated with (1) CK, sterile TSB-s without antibacterial substances; (2) T, sterile TSB-s with 1/2 MIC PLA, and then incubated at 37 ℃ for 6 h. After incubation, the cell suspensions were centrifuged at 8000 g for 3 min to remove the culture medium. The obtained cell pellets were immediately inactivated with liquid nitrogen and temporarily stored at − 80℃ before the following RNA extraction.

The total RNA of *Vp*17802 was extracted by TRIzol reagent (Invitrogen, Carlsbad, USA). Briefly, the bacterial cells were fully ground in liquid nitrogen and the intracellular substances were released from bacterial cells by 1 mL TRIzol reagent. Two hundred microliters of chloroform (Guangzhou Chemical Reagent Factory, Guangzhou, China) was added to separate from the DNA and proteins at 12,000 rpm for 10 min. Then, the upper aqueous phase was taken and 1 mL equal volumes of isopropanol (75%, v/v, Guangzhou Chemical Reagent Factory, Guangzhou, China) were added to precipitate total RNA under vacuum. The quality and integrity of total RNA were assessed using an Agilent 2100 Bio-analyzer (Agilent Technologies, Palo Alto, USA) and RNase-free agarose gel electrophoresis. After total RNA extraction, the ribosomal RNA was removed by Ribo-ZeroTM Magnetic Kit (Epicentre, Madison, USA), and the mRNA was fragmented into short fragments with 200–700 nt using a fragmentation buffer. The mRNA fragments were reversed transcription into cDNA with random hexamer primers, and the second strand of cDNA was synthesized with dNTP to promote DNA polymerase I. The synthesized cDNA fragments were purified using a QiaQuick PCR extraction kit (Qiagen, Venlo, the Netherlands) and then subjected to end repair, the ligation of poly (A), and sequencing adapters. The ligated cDNA fragments were size-screened by the agarose gel electrophoresis. The selected cDNA fragments were amplified and sequenced using Illumina HiSeq2500 by Gene Denovo Biotechnology Co. (Guangzhou, China).

### Read alignment and normalization of gene expression levels

The high-quality reads were conducted using in-house Perl scripts to remove adapters or low-quality bases. The reads containing adapters, more than 10% of unknown nucleotides, and more than 50% of low quality (*Q*-value ≤ 20) bases) were removed. Short read alignment tool Bowtie2 was used to exclude the reads mapped to the rRNA database. The remaining clean reads were then mapped to the *Vp*17802 RIMD 2210633 (NCBI accession: NC_004603.1) using TopHat2. The gene expression levels were normalized using StringTie software. The gene expression abundance was quantified using fragments per kilobase of transcript per million (FPKM) method.

### Differentially expressed genes and enrichment analysis

Bioconductor edgeR package (http://www.rproject.org/) was used to identify differentially expressed genes (DEGs) between the samples. The criteria of significant differential expression were FDR ≤ 0.05 and the absolute value of log2 (FC) ≥ 2. Significantly enriched GO terms and KEGG pathways were obtained with the criteria of an adjusted *p*-value ≤ 0.05. The gene ontology (GO) was applied to define and standardize the function of genes and proteins, including biological process (BP), molecular function (MF), and cellular component (CC). All the expressed genes were mapped to the gene ontology database (http://www.geneontology.org/), and the number of genes mapped to each GO term was calculated. The Kyoto Encyclopedia of Genes and Genomes (KEGG) (http://www.genome.jp/kegg) is the core of the database that was significantly enriched in DEGs compared with the whole genomics defined.

### Data analysis

Data are presented as means ± SDs from at least three independent experiments. Statistical significance was determined using a *t*-test and Waller–Duncan multiple comparison test using GraphPad Prism 5 to analyze the experimental data. Independent sample.

## Results

### The MIC and time-kill curve

**Fig. 1:**
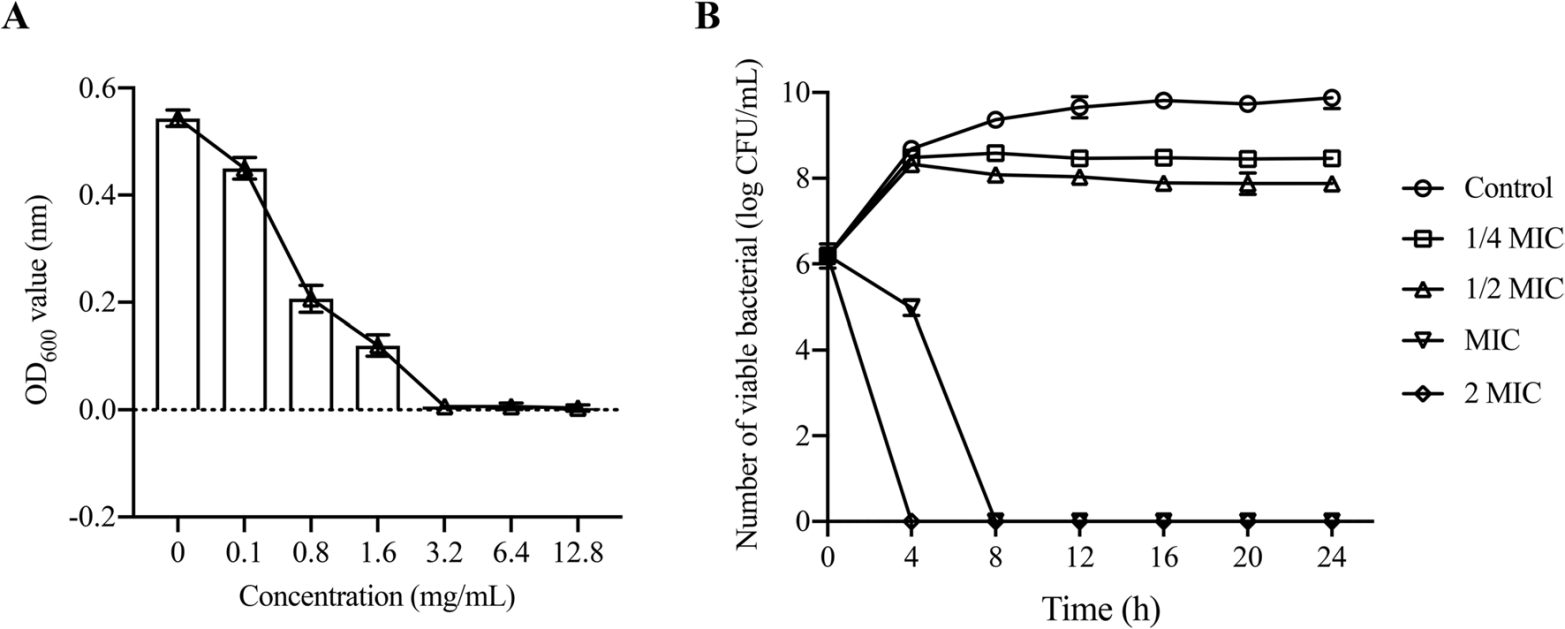
**A** The OD_600_ of the *Vp*17802 culture with different concentrations of PLA. **B** Time-kill curve of *Vp*17802 treated by PLA. Control: the sterile TSB-s without PLA; 1/4MIC, 0.8 mg/mL PLA; 1/2MIC, 1.6 mg/mL PLA; MIC, 3.2 mg/mL PLA; 2MIC, 6.4 mg/mL PLA

The MIC of *Vp*17802 treated with different PLA concentrations after 24 h is summarized in Fig. 1A. Compared to the control group, the OD_600_ of *Vp*17802 was reduced when exposed to PLA of different concentrations with the increase of incubate time. Besides, 3.2 mg/mL PLA could reduce OD_600_ of *Vp*17802 by nearly 0, which can be considered as the MIC. This MIC was consistent with our previous studies (Fang et al. 2021). In addition, the viable bacterial were increased in 1/4MIC and 1/2MIC within 4 h; however, the viable bacterial of MIC and 2MIC groups were completely inactivated (Fig. 1B). These results demonstrated that the antibacterial activity of PLA was found to be in a dose-dependent manner. Moreover, 1/4 MIC and 1/2 MIC of PLA were inactivated gradually after 4 h.

### PLA-induced bacterial cytoplasmic material leakages

The cell membrane destroyed will lead to membrane depolarization, membrane destruction, and membrane pore formation, causing the leakage of cytoplasmic materials. Previous studies have proposed that PLA can produce ROS, which increases membrane damage and leads to *Vp*17802 death (Fang et al. 2021). To further investigate the bacterial membrane damage of PLA in *Vp*17802, the OD_260_ and OD_280_ were analyzed using an ultra-micro ultraviolet–visible spectrophotometer. As shown in Fig. 3C, compared with the control group, the OD value of PLA-treated showed a significant increase, indicating PLA could lead to cytoplasmic leakage in *Vp*17802.

### PLA-induced bacterial ATP level and inhibited swimming ability

**Fig. 2:**
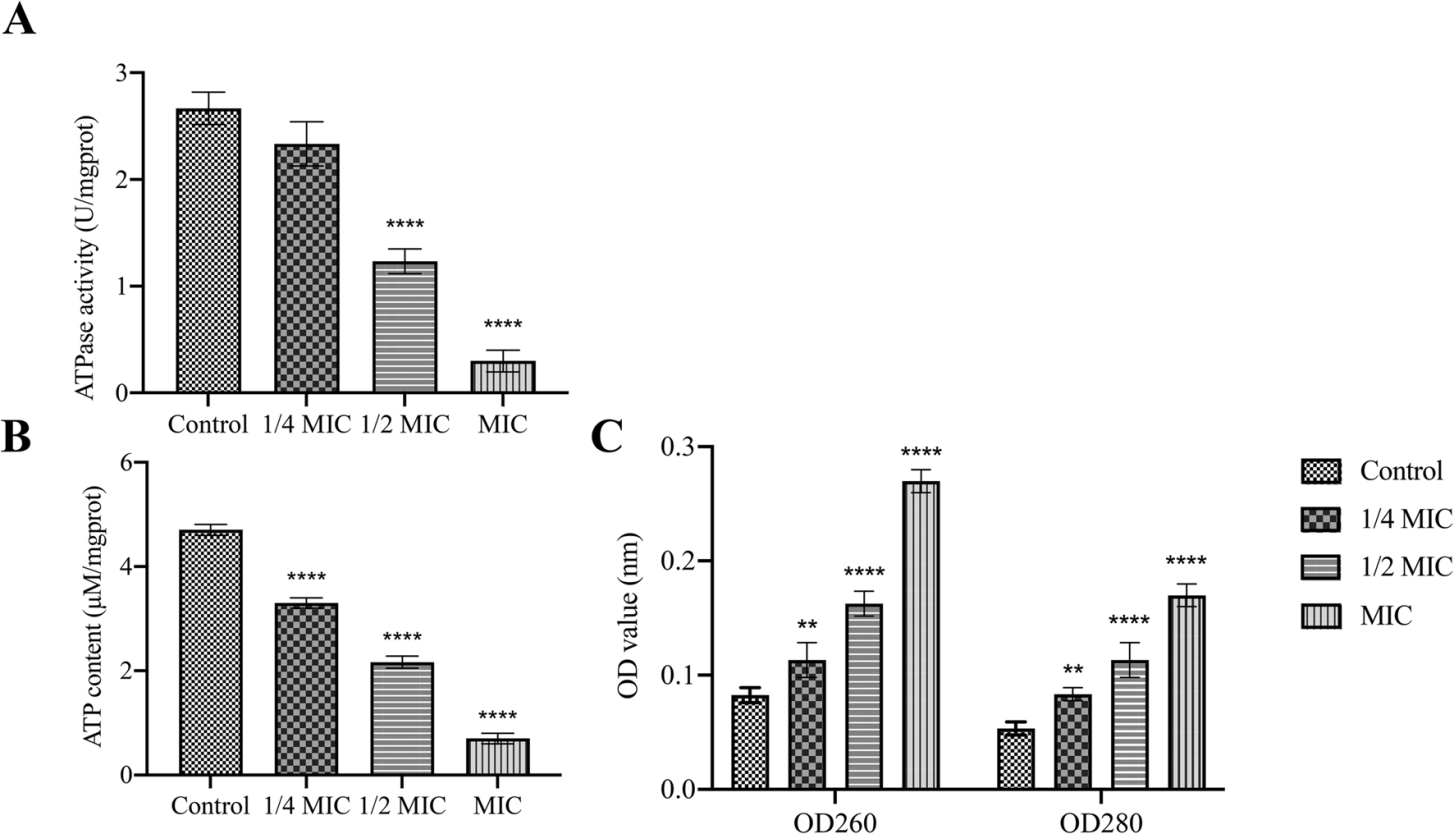
**A** The effect of PLA on intracellular ATPase activity in *Vp*17802. **B** The effect of PLA on intracellular ATP levels of *Vp*17802. **C** The effect of PLA on the OD260 and OD280 of *Vp*17802. All samples were incubated at 37 ℃ for 12 h

**Fig. 3:**
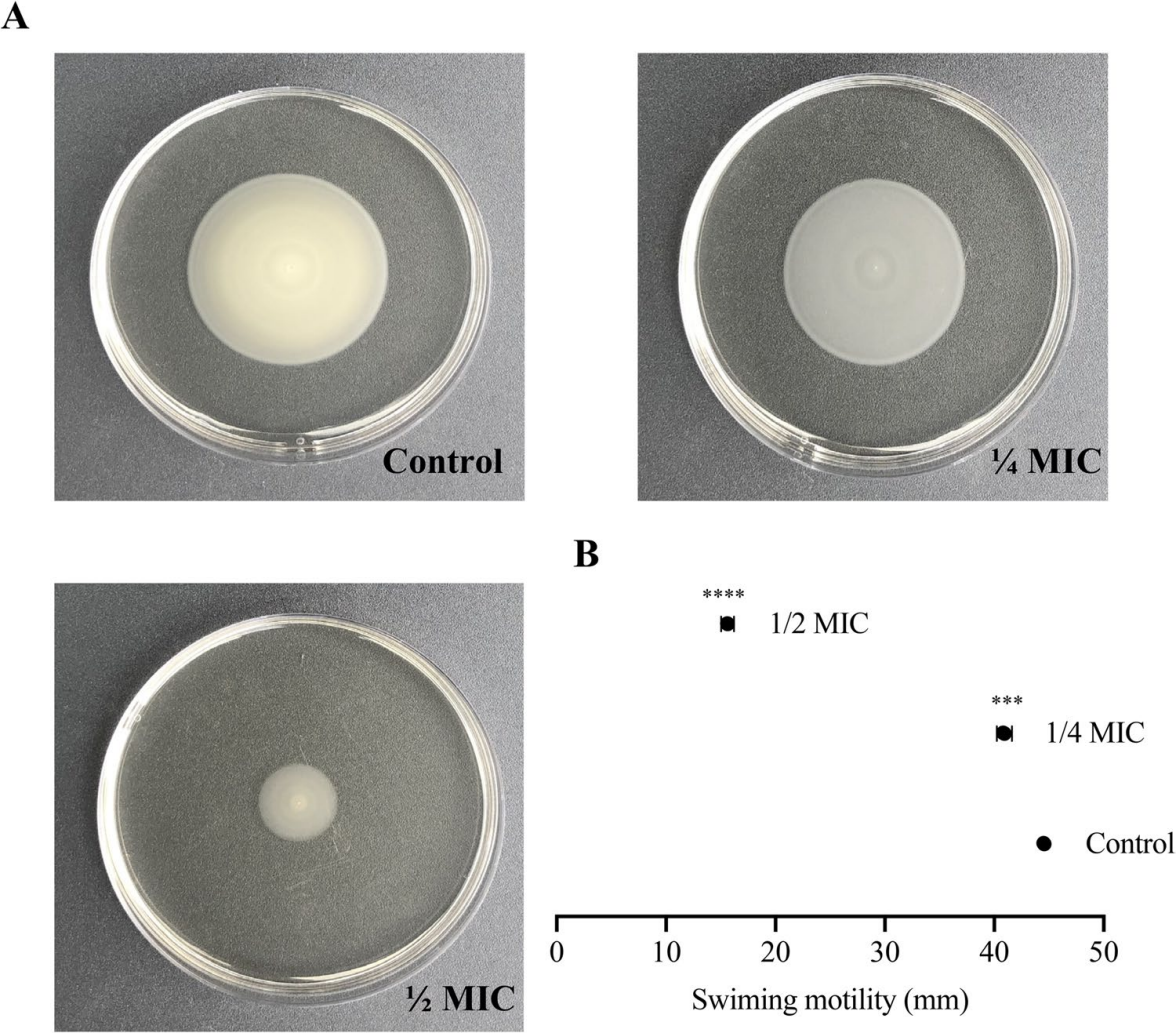
**A** The effect of PLA on swimming ability of *Vp*17802. **B** The diameter of swimming halos

The energy conversion of cells and the secretion of various enzymes depend on the cell membrane. We have demonstrated that PLA can cause *Vp*17802 membrane damage, which may affect the ATP level. As shown in Fig. 2A and B, compared with the control group, the ATPase activity and ATP concentration were significantly reduced by 1/2MIC and MIC of PLA. In addition, our previous studies also showed that *Vp*17802 membrane damage can reduce the ability of cells to form biofilms (Fang et al. 2021). As shown in Fig. 3, we could find that the swimming ability of 1/2MIC and 1/4MIC significantly declined than the control group. It is obvious that the motility of *Vp*17802 was significantly reduced by PLA treatment.

### DEG analysis

**Fig. 4:**
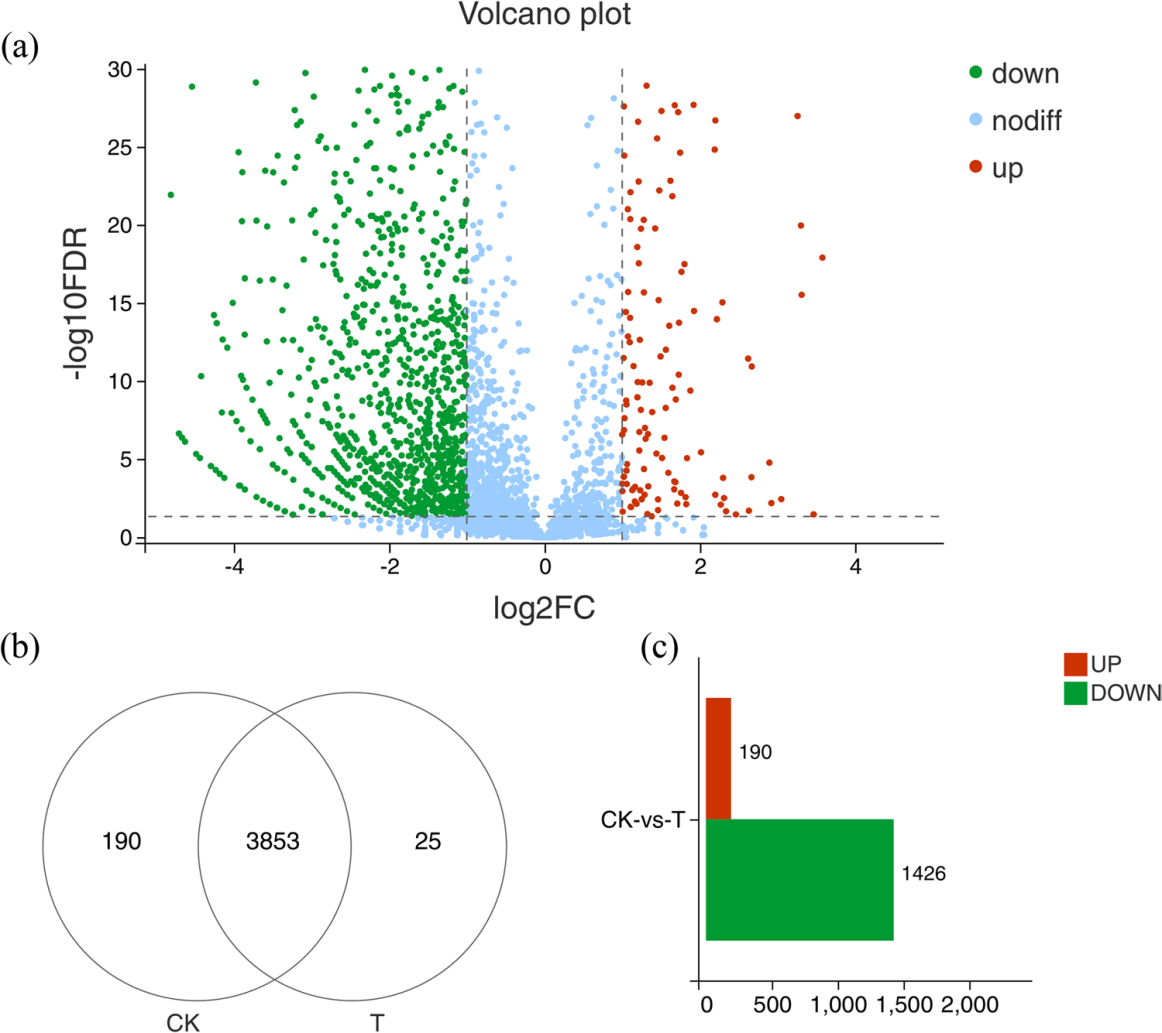
Differentially expressed genes of *Vp *between different treatments. **a** Volcano plots of DEGs of CK-vs-T; **b** Venn diagram of DEGs of CK-vs-T; **c** the number of up-regulated and down-regulated genes of CK-vs-T. CK: the sterile TSB-s without PLA; T: 1/2 MIC PLA. The criteria of DEGs: | log2 (Fold change) |≥ 2, FDR ≤ 0.05

Transcriptomic profiling experiments demonstrated that 1616 DEGs were identified, among which 190 genes were up-regulated and 1426 were down-regulated (Fig. 4c). The Venn diagram shows the number of DEGs of different comparisons (Fig. 4b). Of these, 190 genes were specifically expressed only in the CK group, while 25 genes were differentially expressed only in the T group. The results show that multiple layers of response mechanism may be applied by *Vp*17802 under PLA stress. Further study of the inhibition mechanism from GO and KEGG results is necessary.

### GO enrichment analysis

**Fig. 5:**
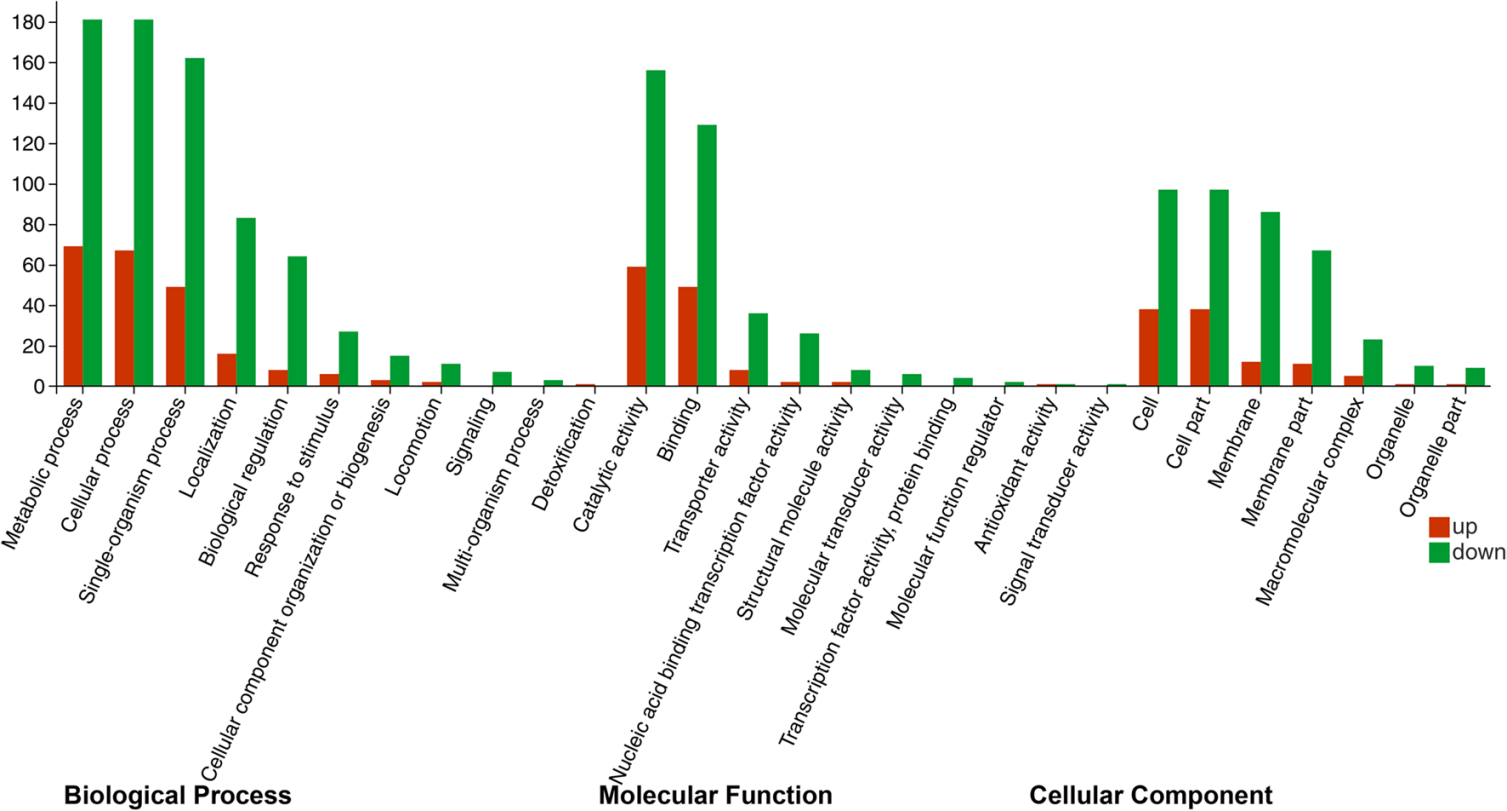
The significantly enriched gene ontology annotations in the GO terms BP, MF, and CC. CK: the sterile TSB-s without PLA; T: 1/2 MIC PLA

The GO enrichment analysis of DEGs was included biological processes, cellular components, and molecular functions. As shown in Fig. 5, BP of CK-T was enriched with a total of 955 differentially expressed genes, mainly involving metabolic process (up, 69; down, 181), cellular process (up, 67; down, 181), organism process (up, 49; down, 165), localization (up, 16; down, 83), and biological regulation (up, 8; down, 64). MF of CK-T was enriched with a total of 490 differentially expressed genes, mainly involving catalytic activity (up, 59; down, 156), binding (up, 49; down, 129), and transporter activity (up, 8; down, 36). CC of CK-T was enriched with a total of 498 differentially expressed genes, main involving cell (up, 38; down, 97), cell part (up, 38; down, 97), membrane (up, 12; down, 86), and membrane part (up, 11; down, 67). Compared with the CK group, the biological process of citric acid and tricarboxylic acid metabolism and the cellular component of cellular ribosomes and ribonucleoprotein complexes were significantly enriched in *Vp*17802 treated with PLA (*Q*-value ≤ 0.05). The results demonstrated that PLA exhibited an effect on the transcription of *Vp*17802 mainly altering its BP and CC.

### KEGG analysis

**Fig. 6:**
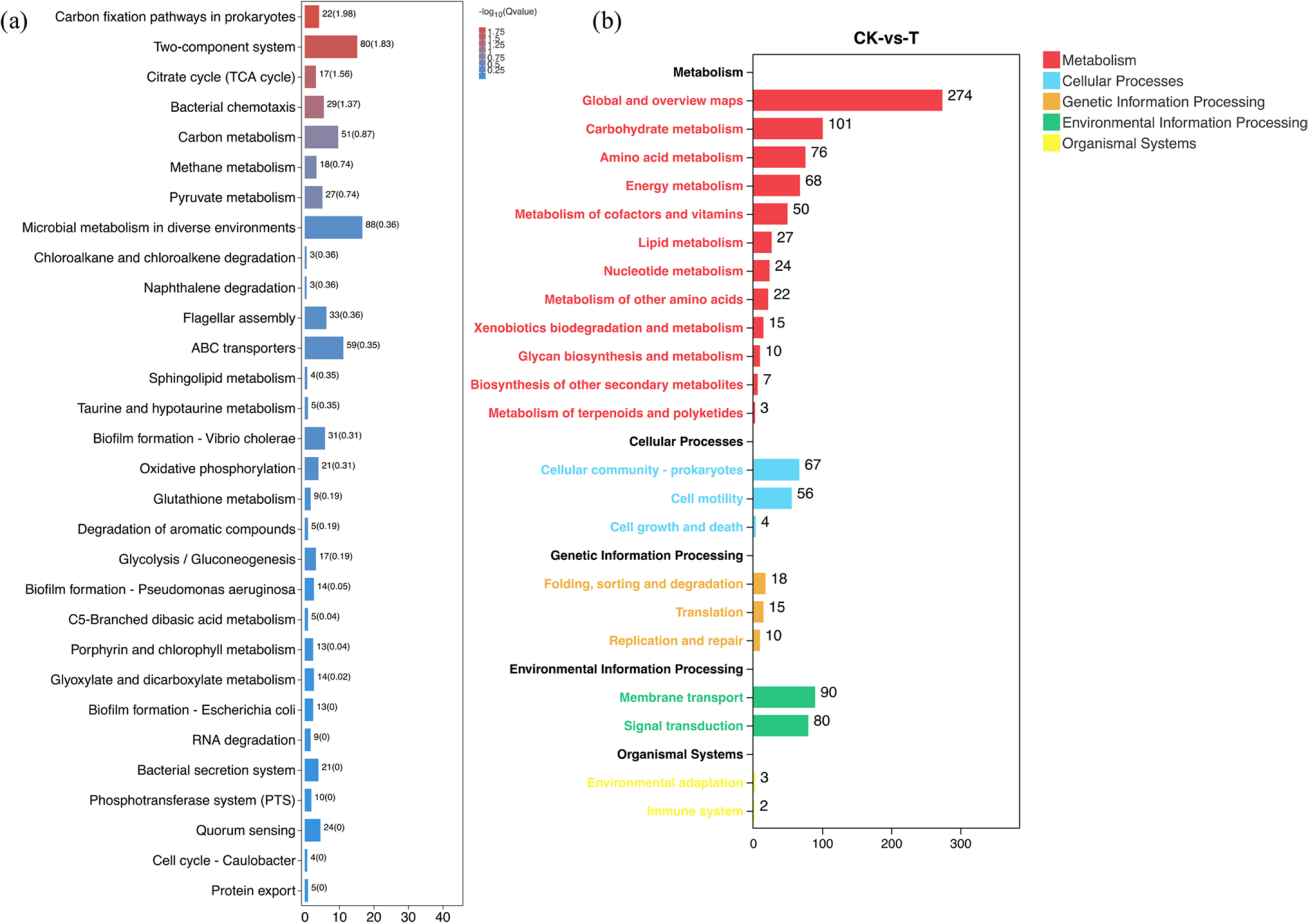
The significantly enriched KEGG pathways of CK-vs-T. CK: the sterile TSB-s without PLA, T: 1/2 MIC PLA

The KEGG pathways analysis of DEGs was assisted to further analyze and understand and explore the antibacterial mechanism of PLA in *Vp*17802. In the results, the metabolism, cellular, genetic information processing, environmental processing, and organismal systems were identified under the PLA treatment in *Vp*17802 (Fig. 6b). Meanwhile, the top seven significant KEGG pathways were enriched, including carbon fixation pathways in prokaryotes, two-component system, the tricarboxylic acid (TCA) cycle, bacterial chemotaxis, carbon metabolism, methane metabolism, and pyruvate metabolism (Fig. 6a). The carbon fixation pathways, two-component system, citrate cycle, bacterial chemotaxis, and carbon metabolism were significantly enriched in *Vp*17802 treated with PLA (*Q*-value ≤ 0.05).

## Discussion

### Antibacterial effect of PLA against *Vp*17802

Studies have shown that the MIC of PLA against *Vp*17802 is 3.2 mg/mL, and other research also revealed the MIC of PLA against bacterial pathogens is 1.5 to 5.0 mg/mL (Ning et al. 2017). These results elucidate that PLA has efficient antibacterial activity on *Vp*17802. It was previously found that intracellular ROS level in *Vp*17802 was increased by PLA-treated (Fang et al. 2021), and a large amount of cytoplasmic material leakages further confirmed that PLA caused oxidative damage to cells and affected bacterial membrane integrity (Fig. 2C). Fang et al. (2021) have also reported that damage of the membrane of *Vp*17802 was treated by PLA. The change in bacterial ATPase activity after PLA treatment also supports these results (Fig. 2A and B). The bacterial membrane was destroyed, which could reduce the ATP synthesis, resulting in a decrease in ATPase activity. In addition, Wadhwa and Berg (2021) have shown that the ATPase activity is related to bacterial motility, which is able to reduce the bacterial swimming ability.

### Transcriptomics analysis by RNA-seq

#### Impairing the energy metabolism

**Fig. 7:**
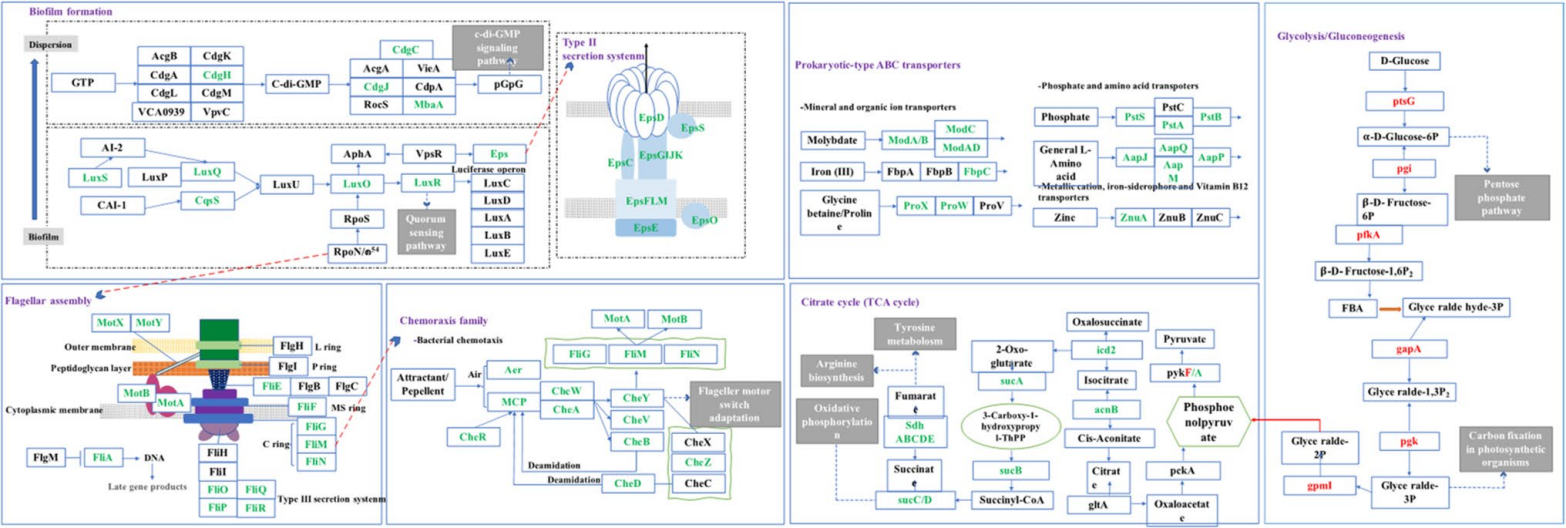
The responses of *Vp*17802 against PLA in 1/2MIC. Genes colored in red or green represent a higher or lower level than the control

The related pathway of tyrosine metabolism, arginine biosynthesis, and oxidative phosphorylation in the tricarboxylic acid (TCA) cycle has been significantly regulated (Fig. 6). The TCA cycle, known as the citrate cycle, is the most important part of supplying energy for the metabolic hub of a cell. The NADP-dependent isocitrate dehydrogenase (*icd2*) is a key enzyme involved in cell metabolism and energy generation through the TCA cycle, which can regulate the oxidative respiration and normal operation of cells. It has been demonstrated that *icd2* has reducing power to resist oxidative damage, and product virulence factors to adapt to the change of external environment (Chiang et al. 2017; Lv et al. 2016). In mycobacteria, the *icd2* is responsible for carbon flux through the oxidative TCA cycle to ensure a balance between energy production and precursor biosynthesis by the glyoxylate shunt enzyme isocitrate lyase (Sharmistha et al. 2005). We found that PLA was able to down-regulate the expression of *icd2*, which means the energy metabolism of *Vp*17802 was destroyed (Fig. 7).

Meanwhile, the 2-oxoglutarate dehydrogenase E1 component (*sucA*) and 2-oxoglutarate dehydrogenase complex dihydrolipoyllysine-residue succinyltransferas (*sucB*) are important enzymes to convert 2-Oxo-glutarate to Succinyl-CoA in the TCA cycle, which were down-regulated by 2.4 log2 (FC) and 2.51 log2 (FC), respectively. The ketoglutarate dehydrogenase gene cluster *sucABCD* and the succinyl-CoA synthase gene cluster *sdhABCDE* were down-regulated by more than 2 log2 (FC) (Table S1). These results further indicate that PLA is able to impact the energy metabolism of *Vp*17802 and inhibit cell growth. The aconitate hydratase B (*acnB*) was down-regulated by 1.53 log2 (FC). Several researches have shown that the *acnB* mutations can lead to decreases in oxidative respiration and increases in antibiotic tolerance, while the changes of the *sucC* do not affect (Barton et al. 2021).

The pyruvate kinase of *pykF* and *pykA* was significantly regulated which affected the degradation of glucose into pyruvate (Table S1). The *pykF* was up-regulated by 1.35 log2 (FC), while the *pykA* was down-regulated by 1.06 log2 (FC). The studies have reported that *pykF* is a key upstream kinase to catalyze phosphoenolpyruvate (PEP) producing ATP and promoting energy metabolism causing the sensitivity of drug-resistant strains (Zuye et al. 2022). Abdelhamid et al. (2019) have demonstrated that *pykA* plays the dominant role in glycolysis, which is relevant for *Pseudomonas aeruginosa* virulence, and it is able to influence bacterial pathogenicity. In addition, the glucose-specific IIB component (*ptsG*) was up-regulated by 2.00 log2 (FC) in the glycolytic pathway; likewise, the *gapA* encode glyceraldehyde 3-phosphate dehydrogenase was up-regulated by 2.25 log2 (FC). We conclude that the up-regulated gene in the glycolytic pathway may maintain the normal physiology of *Vp*17802, but the treatment with PLA ultimately causes unbalanced metabolic in the TCA cycle.

### Impeding the prokaryotic-type ABC transporters

ABC transporters have been identified as membrane-bound proteins, which use ATP hydrolysis-generated energy to complete transmembrane transport. The substances were transported by the ABC transport system, including minerals, organic ions, phosphates, amino acids, metallic cations, iron-siderophores, vitamins, carbohydrates, and proteins. It is important to mediate the uptake of bacterial nutrients, and the discharge of metabolites and toxic substances. This system functions significantly altered 14 genes that were down-regulated mainly related to mineral and organic ion transporters (*modABCD*, *fbpC*, *proXW*), phosphate and amino acid transporters (*pstSAB*, *aapJQMP*), and metallic cation, iron-siderophore, and vitamin B_12_ transporters (*znuA*) (Fig. 7).

The ions of iron and zinc are a class of mineral and organic ions, which can be used as important prosthetic groups to inhibit oxidative stress, prevent the production of reactive oxygen species, and reduce cytotoxicity in bacteria. Among them, iron ions are also involved in the tricarboxylic acid cycle and DNA synthesis. On the contrary, zinc ions play a role in cellular metabolism, defense, repair, and other processes by combining with proteins. According to Fig. 2, PLA is able to reduce ATP levels in *Vp*17802. Based on the result of RNA-seq, the *fbpC* was notably down-regulated by 1.34 log2 (FC), and the encoding superoxide dismutase of *sodAC* was also down-regulated by 3.45 log2 (FC) and 3.12 log2 (FC), respectively. The functional ATPase *fbpC* transported extracellular iron ions into the cells via combing with *fbpB* to form a ferroportin. Moreover, the *pstS* was notably down-regulated by 1.25 log2 (FC), which hindered the expression of *pstA* and further caused the down-regulation of *pstB*. The bacterial resistance was reduced when there were low expression levels of *pstB*. The metabolism and balance of phosphorus were controlled by phosphate and amino acid transports, in which *pstS* is a peripheral binding protein and *pstB* is an ATP binding protein. Low expression levels of *pstB* would lead to decreased ATP binding sites and ATP hydrolysis, which cannot provide power for phosphate and amino acid transports. In addition, the genes of *aapJQMP* mediated the transport of general *L*-amino acids, which plays a vital role in the metabolic activities of bacteria. In our study, the *aapJQMP* were significantly inhibited which were down-regulated by 3.10 log2 (FC), 3.14 log2 (FC), 2.37 log2 (FC), and 2.94 log2 (FC), respectively. These phenomena suggested that PLA could cause cell membrane damage, affecting the ATPase activity, further blocking organic iron, phosphate, and amino acid transport, causing damage to *Vp*17802.

### Inhibiting biofilm formation

Biofilm formation is related to quorum sensing (QS), flagellar assembly, cell chemotaxis, and bacterial motility. Previous studies have shown that the biofilm formation was reduced by PLA in *Vp*17802 (Fang et al. 2021). According to Table S1, it can be found that genes related to quorum sensing pathway (*luxO*, *luxR*, *luxS*, *luxQ*, and *cqsS*) were down-regulated by 1.04 ~ 2.57 log2 (FC) and genes related to c-di-GMP signaling pathway (*cdgJ*, *dgcH*, *dgcC,* and *mbaA*) down-regulated expression by 1.78 ~ 3.32 log2 (FC). In the QS system, bacteria communicate with other bacteria to adjust the expression of genes regulating their growth, adhesion, migration, and biofilm formation. The QS of *Vp*17802 can produce three kinds of signaling molecules, namely harveyi autoinducer-1 (HAI-1), autoinducer-2 (AI-2), and cholera autoinducer-1 (CAI-1), and the *luxN*, *luxP*, *luxQ*, and *cqsS* are receptor proteins (Lu et al. 2019). When the external environment changes, the expression of quorum sensing signaling molecules is activated to form polysaccharide-protein complexes further forming a biofilm. In addition, the c-di-GMP signaling pathway is also closely related with biofilm formation. c-di-GMP can inhibit the activity of ATPase to adjust the function of *fleQ*, which is the main regulator of flagellar synthesis, thereby inhibiting the flagellar assembly and reducing bacterial motility. c-di-GMP can also regulate the production of exopolysaccharides, affecting the maturation of biofilms (GodePotratz et al. 2011). Wang et al. (2021) found that carvacrol can bind to the regulator gene of *luxR* to block the interaction with signaling molecule C4-HSL and interfere regulatory function of the QS. The results of the transcriptomic analysis also showed that carvacrol reduced the expression of genes related to flagella assembly, chemotaxis, *luxR*-mediated QS, and c-di-GMP. Therefore, treatment with PLA in *Vp*17802 may inhibit the QS signal and c-di-GMP signal transduction, reducing the ability to form biofilm.

Type II secretory system (T2SS) related genes (*epsDEFGIJKLM*) were down-regulated by 1.04–1.86 log2 (FC). T2SS is one of the important virulence factors of gram-negative bacteria, responsible for transporting virulence proteins from the periplasmic space to the outer membrane. The T2SS of bacteria have been studied about *Vibrio cholerae*, *Escherichia coli*, *Pseudomonas aeruginosa*, *Klebsiella*, *Legionella pneumophila*, and *Yersinia enterocolitica*; among them, *Vibrio cholerae* is highly homology with *Vp*17802 (Abendroth et al. 2009). The pseudopilus, outer membrane complex, inner membrane platform, and ATPase consisted of T2SS, which contains 12 to 15 different types of proteins. The pseudopilus includes a large pseudopilus *epsG*, and four small pseudopilus *epsH*, *epsI*, *epsJ*, and *epsK*. A core component of the outer membrane complex is *epsD*, which controls the closure and opening of outer membrane proteins during secretion. The inner membrane is composed of three proteins, *epsL*, *epsM*, and *epsF*. The secretion mechanism of T2SS is that the precursor substance of the effector excises the signal peptide through the Sec system and is further folded and modified to be secreted out of the cell. The proteins secreted by T2SS mainly include protease, pectinase, cellulase, and toxin. Both thermostable direct hemolysin (TDH) and thermostable related hemolysin (TRH) of *Vp*17802 mainly depend on T2SS secretion. In this study, down-regulated expression of T2SS-related genes suggests that PLA affects the secretion of *Vp*17802-associated toxins, thereby reducing virulence (Fig. 7).

A total of 18 DEGs related to flagellar assembly were down-regulated by 1.04 log2 (FC) ~ 3.12 log2 (FC), including fli*ARQPONMGFELIGP* and mot*XAB* (Fig. 7). The polar and lateral flagellar are two types of *Vp*17802, driven by sodium and hydrogen ions, respectively. The flagella of polar and lateral have similar basic elements, mainly composed of basal body, hook, and flagellum. The basal body can be divided into the rotor and the stator. The rotor comprises the C ring of *fliG*, *fliM*, and *fliN* in the cytoplasm and the MS ring of multiple *fliF*s in the plasma membrane. The basal body also contains a P ring (*flgI*) in the peptidoglycan layer and an L ring (*flgH*) in the outer membrane. In addition, the polar flagellar has an H ring (*flgT*) and a T ring (*motX* and *motY*). The stator complex is composed of two proteins, the stator of the polar flagellar is *pmoA* and *pmoB*, and the stator of the lateral flagellar is composed of *motA* and *motB*. In this study, *pmoA*, *motB*, *motY*, and *motX* were down-regulated by 1.26 log2 (FC), 1.22 log2 (FC), 2.11 log2 (FC), and 1.59 log2 (FC), respectively, indicating that the polar flagellar and lateral flagellar of *Vp*17802 have been affected. According to Fig. 3, PLA is able to reduce cell motility in *Vp*17802. The rapid rotation of the flagellar endowed the bacteria with motility. However, the genes related to flagellar assembly were significantly down-regulated under the stress of PLA, indicating the dynamic system of the flagellar of *Vp*17802 was inhibited resulting in decreased motility. Studies have shown that the genes related to flagellar assembly in *Vp*17802, such as the gene *fliG* and *mcps*, were also significantly down-regulated by about 0.8 log2 (FC) under the treatment of sub-inhibitory concentrations of benzyl isothiocyanate (Song et al. 2019). Gao et al. (2018) confirmed that the genes *flgA*, *flgC*, and *flgD* were down-regulated in *Vibrio anguillarum* under starvation stress. Qiao et al. (2022) showed that genes related to flagellar assembly (*fliC*, *fliK*, *fliG*, *fliN*, *fliH*, *fliI*, *fliJ*, and *fliA*) were significantly enriched when *Vp*17802 was co-cultured with *Ulva fasciata*, showing a trend of down-regulation. Therefore, the flagellar system is an important target for antibacterial substances to exert antibacterial effects.

When bacteria perceive external stimuli, bacterial chemotaxis guides their trend the beneficial movement. Chemotaxis-related genes such as *mcpU*, *mcpP*, *cheV*, *cheR*, *cheW*, *cheZ*, *cheY*, and *fliN* were significantly down-regulated by 1.01–3.14 log2 (FC), indicating PLA treatment had a significant effect on the chemotaxis ability of *Vp*17802 (Fig. 7). The methyl-accepting chemotaxis proteins (MCPs) is able to sense changes of the external environment and stimulate signal transduction through methylation and demethylation processes. The encoding MCP genes were down-regulated by 1 ~ 3 log2 (FC) in PLA treatment, indicating the perceived ability of cells was decreased. In the bacterial chemotaxis system, the methylation level of MCPs was controlled by the methylation function of the methyltransferase *cheR* and the response regulator *cheB*, affecting the signaling activities of MCPs. *cheW* connects MCPs to a histidine kinase *cheA*, and *cheA* undergoes autophosphorylation reactions to transfer to *cheB*, *cheY*, and *cheV*. As the only phosphatase, *cheZ* accelerates the hydrolysis of phosphate on *cheY*, thereby inhibiting the function of cheY. In addition, the C-terminus of *cheZ* is responsible for binding to *cheY* and catalyzing the dephosphorylation of unbound *cheY-P*, when *cheY-P* binds to *fliM*, the *cheZ* cannot catalyze its dephosphorylation. *cheY* and *cheY-P* are able to control the direction of flagellar rotation by binding to *fliM*. Studies have shown that benzyl isothiocyanate also has the ability to reduce the chemotaxis of bacteria in *Pseudomonas aeruginosa* (Kafantaris et al. 2021). Thus, PLA inhibits the expression of the chemotactic system genes reducing the survival viability of *Vp*17802.

In conclusion, this study revealed the antibacterial mechanism of sublethal PLA against *Vp*17802 using high-throughput transcriptome sequencing technology. The results showed that PLA via multiple pathways inhibited *Vp*17802, including cell transportation, energy metabolism, quorum sensing, flagellar assembly, cell chemotaxis, and biofilm formation. Among them, the expression of amino acids, metal ions, and phosphate-related transporters was reduced by PLA treatment, impacting on transport capacity. The energy metabolism capacity was inhibited, which could cause decreased antioxidant capacity and toxicity. In addition, PLA effectively reduced the chemotaxis, motility, and virulence related to biofilm formation. This study provides new evidence and ideas to reveal the antibacterial mechanical of PLA on *Vp*17802.

## Supplementary Information

Below is the link to the electronic supplementary material.

Supplementary file1 (PDF 106 KB)

## Data Availability

The authors declare that (the/all other) data supporting the findings of this study are available within the article. The transcriptome sequencing raw data have been uploaded to the National Microbiology Data Center (Bio Project: NMDC10018011).
