# Supplementary material for: Transcriptomic analyses of Vibrio parahaemolyticus under the phenyllactic acid stress
Source: Appl Microbiol Biotechnol. 2024 Jan 29;108:180. doi: 10.1007/s00253-024-13024-6 (PMC10824802; doi:10.1007/s00253-024-13024-6)
Supplement: Supplementary file 1 [file 253_2024_13024_MOESM1_ESM.pdf]

Applied Microbiology and Biotechnology

Transcriptomic analyses of *Vibrio parahaemolyticus* under the phenyllactic acid stress

Yilin Lin[a], Meimei Fang[a], Jun Liu[b], Yehui Zhang[b]*, Yigang Yu[a,c]*

[a] South China University of Technology, School of Food Sciences and Engineering, Guangzhou 510640, China;

[b] Sericulture & Agri-food Research Institute Guangdong Academy of Agricultural Sciences, Key Laboratory of Functional Foods, Ministry of Agriculture and Rural Affairs, Guangdong Key Laboratory of Agricultural Products Processing, Guangzhou 510610, China;

[c] South China University of Technology, Research Center of Food Safety and Detection, Guangzhou 510640, China.

* Corresponding author:

Prof. Yigang Yu, email: yuyigang@scut.edu.cn, Tel: +86-20-22236819;

Prof. Yehui Zhang, email: zhangyhgx@163.com, Tel: +86-20-83628150.

Table S-1 The expression of essential genes related to PLA stress in *Vp*17802

Symbol	log2(FC)	P-value	FDR	Description
**Energy metabolism related**				
ptsG	2	1.51E-229	8.30E-228	PTS system, glucose specific IIB component
gapA	2.25	0	0	Glyceraldehyde-3-phosphate dehydrogenase
pykF	1.35	6.06E-110	1.98E-108	Pyruvate kinase
pykA	-1.06	1.33E-12	6.20E-12	Pyruvate kinase
acnB	-1.53	0	0	Bifunctional aconitate hydratase 2/2-methylisocitrate Dehydratase
icd2	-1.16	4.38E-100	1.27E-98	NADP-dependent isocitrate dehydrogenase
sucA	-2.4	0	0	2-oxoglutarate dehydrogenase E1 component
sucB	-2.51	0	0	2-oxoglutarate dehydrogenase complex Dihydrolipoyllysine-residue succinyltransferase
sucC	-2.51	0	0	ADP-forming succinate--CoA ligase subunit beta
sucD	-2.4	2.44E-244	1.53E-242	Succinate--CoA ligase subunit alpha
sdhA	-2.5	0	0	Succinate dehydrogenase flavoprotein subunit
sdhB	-2.86	2.44E-311	0	Succinate dehydrogenase iron-sulfur protein
sdhC	-4.19	3.01E-177	1.36E-175	Succinate dehydrogenase cytochrome b556 subunit
sdhD	-2.89	7.44E-73	1.66E-71	Succinate dehydrogenase hydrophobic membrane anchor
sdhE	-2.4	3.51E-12	1.60E-11	Succinate dehydrogenase assembly factor 2
**ABC transportor related**				
modD	-1.36	1.89E-08	6.71E-08	Molybdate ABC transporter ATP-binding protein
modC	-1.89	2.37E-21	1.69E-20	Molybdenum ABC transporter ATP-binding protein
modB	-2.42	3.47E-16	2.02E-15	Molybdate ABC transporter permease subunit
modA	-2.07	8.70E-21	6.07E-20	Molybdate ABC transporter substrate-binding protein
fbpC	-1.34	3.60E-09	1.35E-08	Ferric iron ABC transporter, ATP-binding protein
znuA	-1.58	2.37E-13	1.15E-12	Zinc ABC transporter substrate-binding protein
proW	-4.14	4.49E-14	2.30E-13	Choline ABC transporter permease subunit
proX	-3.7	9.73E-39	1.20E-37	Choline ABC transporter substrate-binding protein

sodA	-3.45	0.007406	0.013993	Superoxide dismutase
sodC	-3.12	1.98E-06	5.93E-06	Superoxide dismutase family protein
pstS	-1.25	2.28E-06	6.74E-06	Phosphate ABC transporter substrate-binding protein
pstA	-1.31	2.09E-15	1.15E-14	Phosphate ABC transporter permease
pstB	-1.31	0.004652	0.009098	Phosphate import ATP-binding protein
aapJ	-3.1	1.69E-61	3.31E-60	Glutamate aspartate periplasmic binding protein Precursor
aapQ	-3.14	2.06E-13	1.01E-12	Amino acid ABC transporter permease
aapM	-2.37	1.26E-06	3.86E-06	Amino acid ABC transporter permease
aapP	-2.94	2.05E-15	1.13E-14	Amino acid ABC transporter ATP-binding protein
**Cell chemotaxis related**				
mcpU	-3.14	2.65E-28	2.43E-27	Methyl-accepting chemotaxis protein
mcpP	-2.55	6.95E-25	5.62E-24	Methyl-accepting chemotaxis protein
cheV	-1.4	7.60E-57	1.39E-55	Chemotaxis protein
cheR	-2.65	4.02E-305	3.35E-303	Chemotaxis protein methyltransferase
cheW	-1.01	5.36E-16	3.10E-15	Chemotaxis protein
cheZ	-1.03	2.61E-35	2.97E-34	Chemotaxis protein
cheY	-1.85	3.45E-34	3.78E-33	Chemotaxis protein
fliN	-1.31	1.03E-08	3.72E-08	Polar flagellar motor switch protein
fliM	-1.03	6.52E-18	4.06E-17	Polar flagellar motor switch protein
**Flagella assembly related**				
fliA	-1.45	1.39E-47	2.19E-46	Polar flagellar RNA polymerase sigma factor
fliR	-1.32	6.08E-10	2.41E-09	Polar flagellar biosynthesis protein
fliQ	-1.65	0.010733	0.019616	Polar flagellar biosynthesis protein
fliP	-1.43	2.36E-11	1.02E-10	flagellar type III secretion system pore protein
fliO	-1.04	9.87E-05	0.000244	flagellar biosynthesis protein
fliN	-1.31	1.03E-08	3.72E-08	Polar flagellar motor switch protein
fliM	-1.03	6.52E-18	4.06E-17	Polar flagellar motor switch protein
fliG	-1.34	5.04E-25	4.10E-24	Polar flagellar motor switch protein
fliF	-1.15	2.16E-24	1.73E-23	Flagellar M-ring protein

fliE	-1.82	1.39E-14	7.30E-14	Polar flagellar hook-basal body complex protein
fliL	-2.1	9.55E-21	6.64E-20	Flagellar basal body protein
fliI	-1.5	4.26E-06	1.22E-05	Lateral flagellar FliI-like assembly ATPase protein
fliG	-1.61	0.00095	0.00209	FliG lateral flagellar motor switch protein
fliP	-3.12	0.006362	0.012167	FliP lateral flagellar protein
motY	-2.11	7.34E-10	2.89E-09	Sodium-type flagellar protein
motX	-1.59	3.65E-12	1.66E-11	Sel1 repeat family protein
motA	-1.26	4.10E-05	0.000106	Polar Na+-driven flagellar motor protein
motB	-1.22	1.55E-07	5.14E-07	Flagellar motor protein
**Biofilm formation related**				
luxO	-2.57	3.22E-15	1.75E-14	Sigma-54-dependent Fis family transcriptional regulator
luxR	-1.04	4.18E-09	1.55E-08	Quorum-sensing regulator OpaR
luxS	-1.47	1.47E-17	8.99E-17	S-ribosylhomocysteine lyase
luxQ	-1.47	2.17E-36	2.54E-35	Response regulator
cqsS	-2.37	3.78E-44	5.30E-43	Response regulator
cdgJ	-1.84	4.39E-20	3.00E-19	Histidine kinase
dgcH	-3.24	1.60E-284	1.26E-282	GGDEF domain-containing protein
cdgC	-1.78	2.91E-12	1.33E-11	GGDEF and EAL domain-containing protein
mbaA	-3.32	1.31E-17	8.08E-17	EAL domain-containing protein
**Type II secretion system related**				
epsD	-1.07	4.56E-32	4.69E-31	General secretion pathway protein
epsE	-1.32	1.18E-57	2.17E-56	General secretory pathway protein
epsF	-1.44	4.82E-37	5.77E-36	Type II secretion system inner membrane protein
epsG	-1.16	8.44E-32	8.63E-31	General secretion pathway protein
epsI	-1.86	3.97E-10	1.60E-09	General secretion pathway protein
epsJ	-1.21	5.64E-12	2.53E-11	Type II secretion system minor pseudopilin
epsK	-1.4	2.44E-22	1.85E-21	General secretion pathway protein
epsL	-1.22	2.48E-27	2.18E-26	Type II secretion system protein
epsM	-1.05	0.000125	0.000304	General secretion pathway protein
